# The cutaneous microbiome in hospitalized patients with pressure ulcers

**DOI:** 10.1038/s41598-020-62918-8

**Published:** 2020-04-06

**Authors:** Luuk A. de Wert, Sander S. Rensen, Zita Soons, Martijn Poeze, Nicole D. Bouvy, John Penders

**Affiliations:** 1 Department of Surgery, Maastricht University Medical Centre, Maastricht, The Netherlands; 2 NUTRIM School for Nutrition and Translational Metabolism, Maastricht University, Maastricht, The Netherlands; 3 Department of Medical Microbiology, Maastricht University Medical Centre, Maastricht, The Netherlands; 4 CAPHRI School for Public Health and Primary care, Maastricht University Medical Centre, Maastricht, The Netherlands

**Keywords:** Microbiology, Medical research

## Abstract

This study investigated whether there are differences in the composition of the cutaneous microbiome of the unaffected skin between patients with pressure ulcers compared with those without pressure ulcers. The cutaneous microbiome of the unaffected skin of 15 patients with sacral pressure ulcers compared to 15 patients without pressure ulcers was analysed. It demonstrated that the inter-individual variation in skin microbiota of patients with pressure ulcers was significantly higher (P = 0.01). The abundance of 23 species was significantly different with *Staphylococcus aureus* and unclassified *Enterococcus* the most abundant species in patients with pressure ulcers. Random Forest models showed that eight species were associated with pressure ulcers occurrence in 81% of the patients. A subset of four species gave the strongest interaction. The presence of unclassified *Enterococcus* had the highest association with pressure ulcer occurrence. This study is the first to demonstrate that the cutaneous microbiome is altered in patients with pressure ulcers.

## Introduction

Pressure ulcers, or decubitus, are an important clinical problem, especially in the elderly, disabled, or those who are bound to a bed or wheelchair. Yearly, over 2.5 million hospitalized patients are treated in the United States for pressure ulcers with estimated costs of 11.0 billion U.S. Dollars^1,2^. Pressure ulcers develop as a result of prolonged mechanical loading on the skin over a bony prominence, mostly at the sacral area. Several internal factors can further contribute to the development of pressure ulcers. For example, increasing age, unconsciousness, urinary and/or bowel incontinence, poor nutritional status, Diabetes Mellitus, paralysis, and cardiovascular diseases have been associated with an increased risk of developing pressure ulcers^3^.

The human skin harbours a large number of microorganisms, the so-called microbiota including bacteria and fungi. A well-balanced cutaneous microbiota is essential in maintaining a healthy skin environment. Indeed, many skin diseases are associated with changes in microbiota composition. For example, an increase in relative *Staphylococcus aureus* abundance plays an important role in the pathophysiology of atopic dermatitis flares, while certain strains of *Propionibacterium acnes* contribute to the development of Acne Vulgaris^4^. In wounds, bacteria impair healing by forming a biofilm, which eventually, may lead to chronic non-healing wounds^5,6^.

In view of the impact of skin bacteria on the development of skin disorders as highlighted above, cutaneous microbiota differences may significantly contribute to the risk of developing pressure ulcers. Such potential differences may offer a new way to identify patients at increased risk for pressure ulcers and can lead to new preventive measures based on modulation of the microbiota as well^7^.

Therefore, the aim of this study was to examine the cutaneous microbiome of the unaffected skin of hospitalized patients with and without sacral pressure ulcers.

## Results

### Study population

Thirty patients were included in this study between July 2015 and August 2015, 15 patients with pressure ulcers (DC group) and 15 control patients (NoDC group). All patients were bound to a bed or chair and admitted to one of the wards of MUMC+. Patients were matched for age, sex, BMI, Diabetes Mellitus, antibiotics use, and medical diagnosis. Baseline characteristics are presented in Table 1. We obtained skin swabs from the intact skin of vertebrae level L3, and not the pressure ulcer in the sacral area itself.

**Table 1:** Baseline characteristics of included participants (n = 30)*.

	DC group (n = 15)	NoDC (n = 15)
Age- years	72.8 ± 8.8	73.2 ± 5.5
Male sex	9 (60.0)	9 (60.0)
Height- centimetres	172.4 ± 9.2	169.9 ± 13.5
Weight- kilogram	78.8 ± 20.0	76.0 ± 12.6
BMI- kg/cm^2^	26.2 ± 5.5	26.7 ± 4.0
Antibiotics use	9 (60.0)	8 (53.3)
**Diagnosis of hospital admission**		
Orthopaedic	7 (46.7)	6 (40.0)
Gastro-intestinal	4 (26.7)	6 (40.0)
Cardiovascular	3 (20.0)	2 (13.3)
Respiratory	1 (6.7)	1 (6.7)
**Comorbidities**		
Diabetes Mellitus	3 (20.0)	3 (20.0)
Neuropathy	1 (6.7)	0
Paraplegia	1 (6.7)	0

Data are presented as mean ± SD or number (%).

NS = Not significant.

*This number includes the samples of three patients in the control group, which failed during sequencing because of low bacterial DNA yield and which were removed from further analysis.

### Sequencing and taxonomic composition

A total of 5,021,668 paired-end reads were generated. After trimming, quality filtering, and removal of potential chimeric reads, 3,707,991 sequences were retained for downstream analysis and clustered into 2,169 operational taxonomic units (OTUs). Samples from three control participants failed during sequencing, because of low bacterial DNA yield, and were discarded for subsequent analysis. The number of sequences for the remaining samples ranged from 48,952 to 259,953 (median 117,362).

### Taxonomic composition

**Figure 1:**
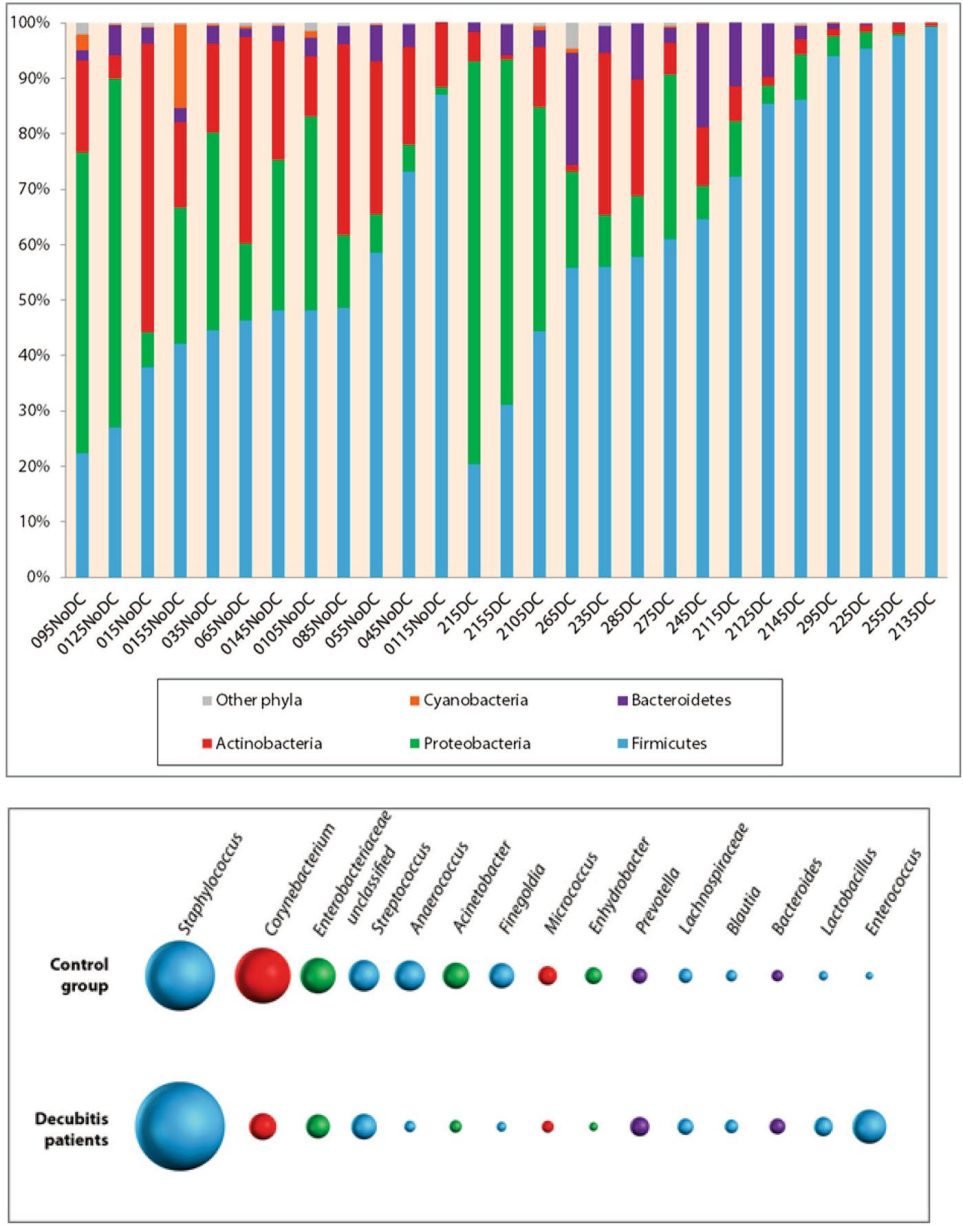
(**A**) Relative abundance of the most predominant bacterial phyla in the control (NoDC) and decubitus group (DC) patients. (**B**) Average proportion of the 15 most abundant bacterial genera in the control group (NoDC) and decubitus group (DC) patients.

In both patients groups, Firmicutes, Proteobacteria and Actinobacteria were the most abundant bacterial phyla, whereas at the genus-level, *Staphylococcus spp*. and *Corynebacterium spp*. predominated (Fig. 1A,B).

### Microbial richness and diversity are not altered in patients with sacral pressure ulcers

**Figure 2:**
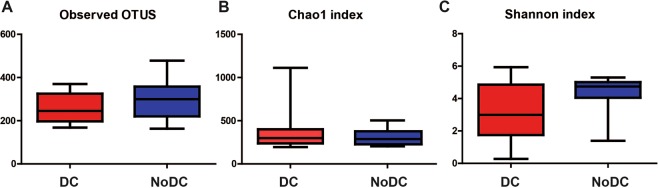
Box and Whisker plots of the Alpha diversity metrics. (**A**) Observed OTUs, P > 0.05 Mann Whitney U test. (**B**) Chao1 index, P > 0.05 Mann Whitney U test. (**C**) Shannon index, P > 0.05 Mann Whitney U test.

The DC and NoDC groups did not show a significant difference with respect to average number of observed OTUs (DC group: 245.0 (168.0–370.0)), NoDC group 299.5 (163.0–478.0)) (Fig. 2A). The estimated richness of the DC group (Chao1, 299.6 (195.1–1112)) and No DC group (Chao1, 287.8 (204.0–504.3)) did not differ significantly (Fig. 2B). The Good’s estimator suggested >99.95% coverage for all samples included in the present study, indicating that only an additional five OTUs would have been found if the sequencing depth were increased with 1,000 reads. Also, microbial diversity as estimated by the Shannon diversity index was not statistically significantly different in skin swabs between the DC and NoDC group, although a larger variation in microbial diversity was observed in the DC group (Fig. 2C). Altogether these results indicate that the microbial richness and evenness are not affected in patients with sacral pressure ulcers.

### The skin microbial community structure is related to disease occurrence

Next, we assessed the dissimilarity in the microbial composition (beta-diversity) of the skin using the Bray-Curtis and the weighted Unifrac distances. Unifrac distances are based on the fraction of branch length shared between two communities within a phylogenetic tree constructed form the 16S rRNA gene sequences from all communities being compared. A relatively small UniFrac distance implies that two communities are compositionally similar, harbouring lineages sharing a common evolutionary history^8^.

**Figure 3:**
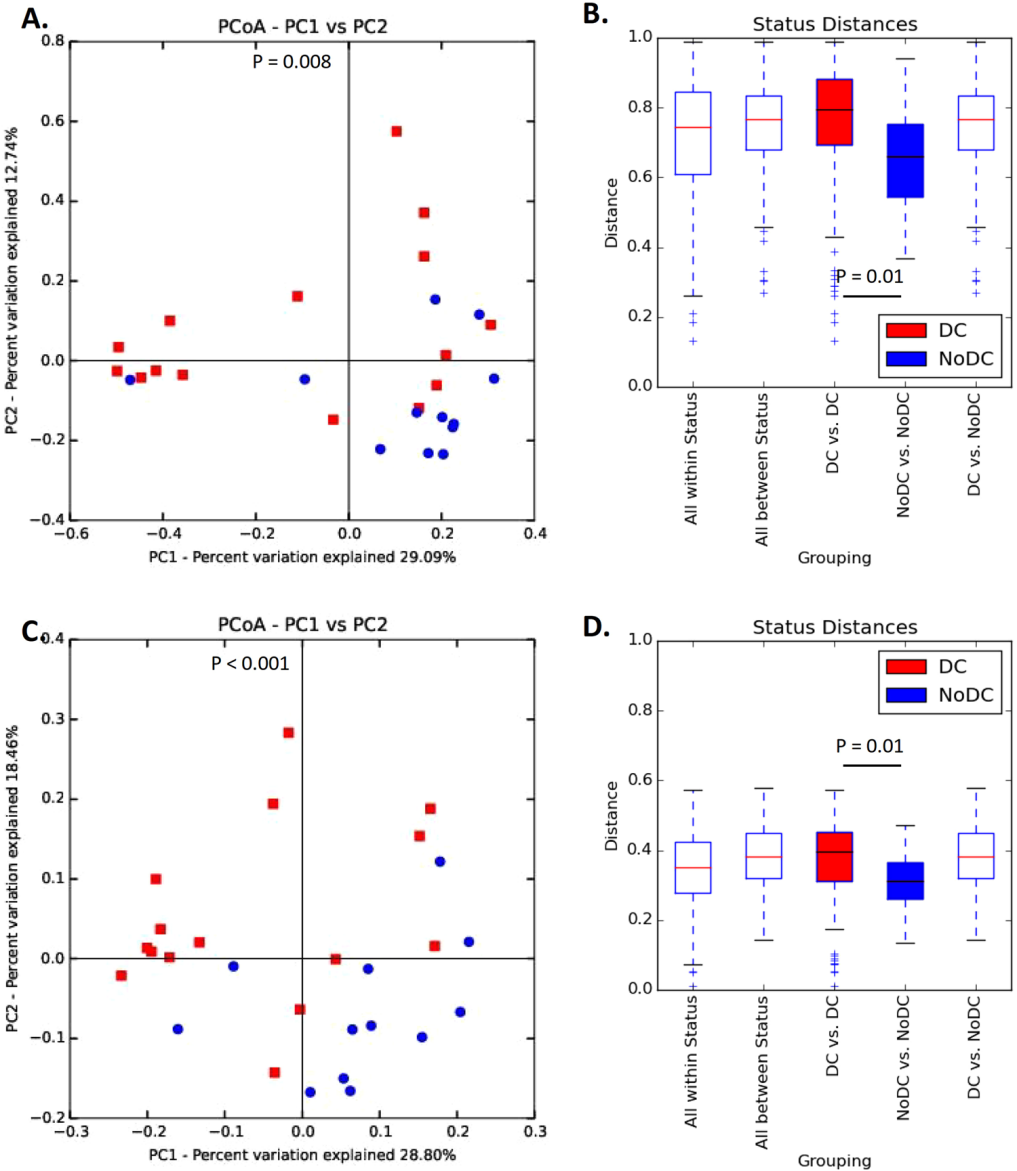
Microbial community structure  (at OTU-level) for patients with and without sacral pressure ulcers. (**A**) Principal Coordinate Analyses (PCoAs) based on Bray-Curtis distance coloured according to disease occurrence (DC group patients in red and NoDC group patients in blue). Variation explained by the principal coordinates: PC1 (29.1%); PC2 (12.7%). (**B**) Box and Whisker plots of the within and between group Bray-Curtis distances. (**C**) Principal Coordinate Analyses (PCoAs) based on weighted Unifrac distance coloured according to disease occurrence (decubitus group patients in red and control group patients in blue). Variation explained by the principal coordinates: PC1 (28.8%); PC2 (18.5%). (**D**) Box and Whisker plots of the within and between group weighted Unifrac distances.

Visualization of Bray-Curtis and weighted Unifrac dissimilarities using PCoA, indicated that many, but not all patients in the DC group clustered apart from the NoDC group patients (Fig. 3A,C). Separation was statistically significant as tested by permutational multivariate analysis of variance on these distance metrics (p = 0.008 and p < 0.001, respectively). This implies that the skin microbial communities in part of the patients with sacral pressure ulcers are structurally and significantly different from the microbial communities in patients without pressure ulcers.

Moreover, the dissimilarity in the microbial community structures in skin swabs within the DC group was significantly larger than the dissimilarity within the NoDC group. This indicates that the inter-individual variation of the skin microbiota of patients without sacral pressure ulcers is significantly smaller as compared to the inter-individual variation in the cutaneous microbiome of patients with sacral pressure ulcers (Fig. 3B, P = 0.01 for Bray-Curtis and Fig. 3D, P = 0.01 for weighted Unifrac).

### Species abundance

**Figure 4:**
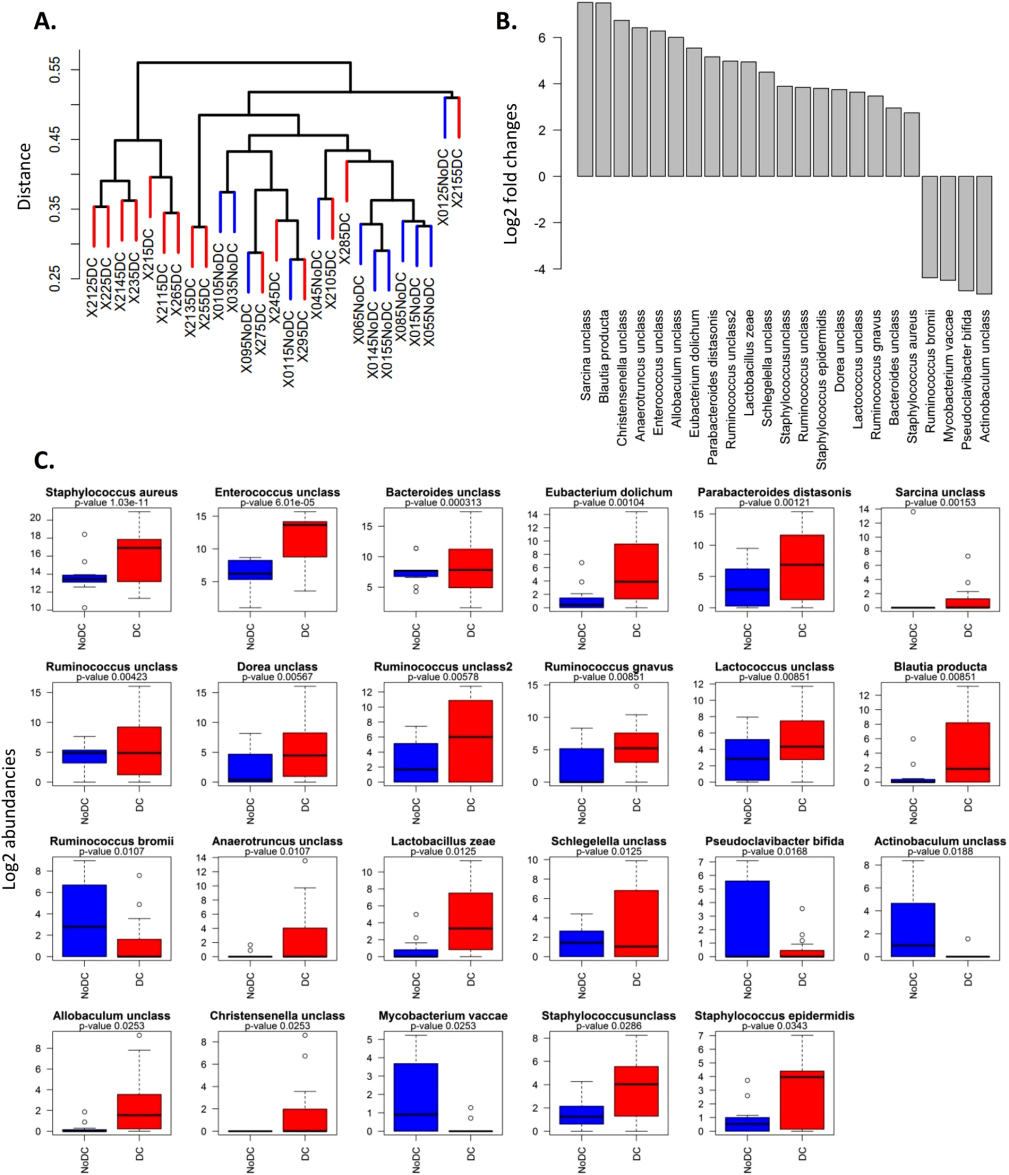
Differential species abundancies between DC and NoDC group patients. (**A**) Cluster diagram. (**B**) Log2 fold changes of the species with significantly different abundance in DC vs. NoDC group patients. (**C**) Boxplots of log 2 abundancies of these 23 species.

Hierarchical clustering based upon the species level relative abundance revealed distinct clusters for half of the patients with and without pressure ulcers (Fig. 4A). A few patients in the DC group were closer to disease free patients of the NoDC group based on the overall species abundancies. We next focussed on the species that distinguish patients with and without pressure ulcers (DC and NoDC group). The abundance of 23 species was significantly different, ranging from a 184-fold more abundant presence (log2 fold change of 7.5) to a 34-fold less abundant presence (log2 fold-change of 5.1) in the DC group. *Staphylococcus aureus* and unclassified *Enterococcus* were the most abundant species in the DC group. (Fig. 4B,C). Unclassified *Corynebacterium* and unclassified *Streptococcus* were also very prevalent in the dataset, but did not show differential abundance. Microbiome abundance tables contain many zeros (Supplementary File 1, Fig. 2). The median abundance of a species is zero, the mean log2 abundance 2.1. The prevalence of any of the 23 differentially abundant (DA) species is thus above average. In particular the ten highest ranked DA species based on p-values are highly abundant. The DA species with 0.01 < p-value <0.05 are low-abundant ones. Despite the low abundance on average, the presence of these species in certain patients might be distinct between DC or noDC, which we investigate using random forests.

### Random forest analysis

To further test the potential clinical relevance of bacterial species and clinical factors in pressure ulcers, we carried out a Random Forest analysis using the species abundances, resp. clinical data. We found that the clinical data in Table 1, such as sex, antibiotics use, and BMI, were not significantly related to pressure ulcer occurrence. Supplementary Figure 3 (Supplementary File 1) shows that none of the clinical factors has a higher correlation to pressure ulcer outcome than the best random predictor. In addition, when all clinical data were taken together, the observed error rate (41%) is not significantly better either than a random predictor in a Fisher test (50%).

**Figure 5:**
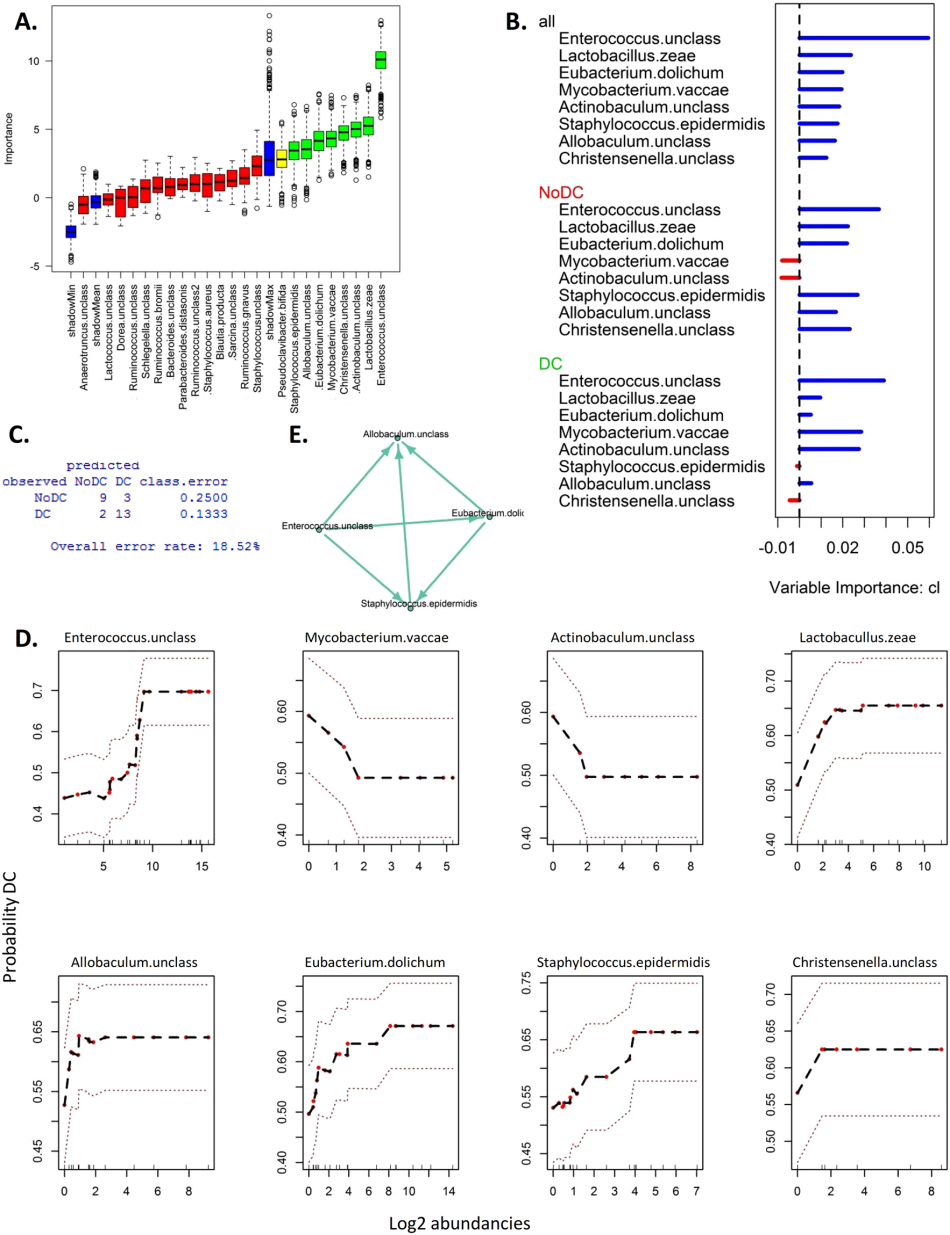
Random Forests with Boruta feature selection using species abundance data. (**A**) The importance of each feature across 1000 repeats is shown compared with the worst, best, and mean randomly generated features. (**B**) Variable importance in the random forest. (**C**) Confusion matrix. (**D**) Effect of species abundance on expected pressure ulcer occurrence in the random forest model. Values on the vertical axis represent the expected probability of a pressure ulcer for a given species, after adjusting for all other predictors. Dashed red lines represent ±2 standard error. The red dots represent samples with corresponding inner lines on the x-axis representing the values of the abundancies. Expected probability based on random classification is 0.44. (**E**) Interaction plot.

Classification based on species abundance, however, showed that eight species are significantly related to pressure ulcer occurrence in the Boruta algorithm (Fig. 5A). These eight species together classified pressure ulcer occurrence correctly in 81% of the patients (error rate of 19%) (Fig. 5C). Figure 5B shows that unclassified *Enterococcus* is also the most important variable for classification as DC and NoDC in a multivariate model. Figure 5D shows the expected probability of having a pressure ulcer as function of species abundance, adjusted for all other species abundances. High abundance of for instance, unclassified *Actinobaculum* is negatively associated with a higher probability of having a pressure ulcer, whereas the abundance of unclassified *Enterococcus* is positively associated with higher probability of having a pressure ulcer.

Interestingly, a subset of four species giving the strongest interactions in an interaction plot (Fig. 5E), unclassified *Enterococcus*, unclassified *Allobaculum, Eubacterium dolichum* and *Staphylococcus epidermidis*, correctly classify pressure ulcer occurrence in 74% of the patients (error rate of 26%). Species tending to split close to the root node have a strong effect on classification, whereas species that split further down the tree have less impact on pressure ulcer occurrence. Species that split close to each other in the tree represent stronger interactions, and thus *together* have greater impact on pressure ulcer occurrence. The source of the arrow in the interaction plot is the more influential species (split closer to the root node). More specifically, the presence of unclassified *Enterococcus* has the highest impact on pressure ulcer occurrence. Since unclassified *Enterococcus*, unclassified *Allobaculum, Eubacterium dolichum* and *Staphylococcus epidermidis* are highly abundant in different patients, a combination of these four species contains additive information leading to better classification of pressure ulcer occurrence.

## Discussion

The cutaneous microbiome plays an important role in providing a healthy environment of the skin, and microbiome alterations influence host-microbe interactions leading to changes in host metabolism and immunity^9^. Therefore, maintaining a well-balanced cutaneous microbiome is essential in preventing the development of skin diseases. To our knowledge, this study is the first to investigate whether there are differences in the cutaneous microbiome of the unaffected skin between hospitalized patients with pressure ulcers compared with those without pressure ulcers. It demonstrates that the microbiota of the unaffected skin in patients with sacral pressure ulcers differs from those without pressure ulcers. Although microbial diversity and richness were not different between patients with or without sacral pressure ulcers, there was an increase in inter-individual variation of the cutaneous microbiome in patients with sacral pressure ulcers. In addition, the abundance of 23 bacterial species on the skin were significantly different in patients with sacral pressure ulcers.

The development of pressure ulcers is the result of prolonged mechanical loading in the form of pressure and shear forces. However, local changes in the skin microclimate have been shown to be a marked risk factor for the development of pressure ulcers in earlier studies^10–12^. In addition, changes in environmental factors influence the skin microenvironment and lead to differences in the composition of the cutaneous microbiome^13^. Cell damage caused by mechanical loading triggers an immune response by the release of damage associated molecular patterns (DAMPs) to initiate the process of cell repair. Several studies have shown that the cutaneous microbiota acts as a modulator of this immune response by the production of metabolites or pathogen associated molecular patterns. For example, a mouse study demonstrated that mice treated with oral vancomycin display cutaneous microbiota dysbiosis and downregulation of RegIIIy and IL-17, leading to delayed wound healing^14^. It is unlikely that changes in the microbiota directly cause pressure ulcers to develop, but they may play a role in the repair of mechanically damaged skin cells. Possibly, the microbiota influences the ability to recover from mechanically induced skin damage and therefore could be related to pressure ulcer development. Based on the results of the present study we only demonstrate association and cannot draw conclusions about causality.

It is particularly promising that we were able to identify eight species that were associated with pressure ulcer occurrence in random forest models. A high abundance of one of defined six species was associated with a higher prevalence of sacral pressure ulcers, suggesting that patients with a higher abundance of one of these species might already be at higher risk for developing sacral pressure ulcers. Combining information on the abundance of several species further improved the classification of the presence of a pressure ulcer. Therefore, assessing pressure ulcer correlates based on a multivariable approach with microbial species as biomarkers might provide a potential novel method to pressure ulcer risk assessment in hospitalized patients in the future.

An analysis of wound microbiota performed by Wolcott and colleagues^6^ demonstrated a high abundance of *Staphylococcus aureus, Staphylococcus epidermidis*, and *Pseudomonas aeruginosa* in pressure ulcers. It must be noted that some changes in the cutaneous microbiota could also be the result of a pressure ulcer near the sampling site. Indeed, we demonstrated a high abundance of *Staphylococcus aureus and Staphylococcus epidermidis* in our study. However, both species were not associated with pressure ulcer occurrence in our random forest models, so it is most likely that other factors were responsible for the shift in microbiota species.

Interestingly, unclassified *Enterococcus* and *Eubacterium dolichium*, both commensal gut bacteria, contribute most to the random forest model. Furthermore, a higher abundance of several other gut bacteria including *Christensenella, Lactobacillus zeae* and unclassified *Allobaculum* were also associated with a higher occurrence of pressure ulcers. In hospitalized patients, up to 17% have some form of faecal incontinence and it is a daily challenge for healthcare workers to prevent faecal incontinence in patients^15^. Although faecal incontinence is associated with an increased pressure ulcer risk^16,17^, there are also studies with contradictory results^18,19^. However, based on the results of the present study, it could be beneficial to protect the skin against faecal incontinence to prevent the development of pressure ulcers.

On the other hand, a higher abundance of four species was found in patients without pressure ulcers, where two species in particular, unclassified *Actinobaculum* and *Myobacterium vaccae*, were associated with absence of pressure ulcers in the random forest models. Interestingly, Keshavarz Valian and colleagues^20^ demonstrated that the administration of a low dose of *Myobacterium vaccae* protected mice against the development of skin ulcera caused by a parasitic infection with *Leishmania major*. Therefore, there might be a role of specific types of bacteria in protecting the skin against the development of wounds such as pressure ulcers in humans as well.

Immobile patients are at increased risk for pressure ulcer development. All participants in our study were bound to a bed or chair and therefore at increased risk for pressure ulcer development.The cutaneous microbiome is also known to be influenced by several other clinical factors such as BMI, diabetes mellitus^21^, antibiotic use^14^, sex^22^, and age^23^. The results of our study did not reveal any relation of these clinical factors with pressure ulcers, because all patients were matched with comparable control patients. This supports that differences in outcomes in the current study were not likely to be caused by confounding factors. In order to minimize differences in hygienic procedures, patients were also matched with control participants from the same hospital wards.

Other measures to prevent external influences on outcomes included sampling by one member of the research team, sampling in similar weather conditions (July-August), and at approximately the same time every day (01.00–03.00 PM). However, many other factors could contribute to pressure ulcer development and changes in cutaneous microbiome composition, e.g. nutritional status, smoking behaviour, and vasopressor agents.

Some other comments need to be placed with respect to the interpretation of the results. First, we focussed on sacral pressure ulcers while pressure ulcers can develop at other skin areas (e.g. the heels, elbows, hips) as well. This might limit the generalizability to patients who developed pressure ulcers at different sites, because the composition of the cutaneous microbiome depends on the type of microenvironment (dry, moist, sebaceous)^13^. We chose to focus on sacral pressure ulcers, because those are most common in hospitalized patients^24,25^, and are more difficult to prevent compared to pressure ulcers at other anatomical locations.

In addition, we chose to obtain skin swabs from the skin of vertebrae level L3 and not to obtain samples from the sacral area itself, because we wanted to obtain skin samples from the unaffected skin and not from the pressure ulcers themselves. Although we did not obtain skin swabs from the sacral area, it is thought that the type of cutaneous microenvironment of vertebrae level L3 is comparable with those of the sacral area^13^.

Second, this study is limited by the relatively small sample size, but also its cross-sectional design. Despite the small sample size, statistically significant differences were found between the two groups. However, it is impossible to determine if these differences in cutaneous microbiome caused changes in skin physiology that eventually promoted the development of pressure ulcers, or whether the microbial shifts were a consequence of alterations in skin physiology/microenvironment as a result of prolonged mechanical loading. Although pressure ulcers are mainly caused by prolonged mechanical loading on the skin, future prospective studies should assess whether the changes in the cutaneous microbiome prior to the manifestations of pressure ulcers are a risk factor for pressure ulcer development. In addition - although random forest bootstrapping methods have been shown to be nearly identical to cross-validation - if the subcutaneous microbiome is to be used as a biomarker, validation using a larger, independent cohort is required.

Third, a limitation of the present study is the 16S rRNA (V4) hypervariable gene region that has been sequenced to profile skin microbiota. It has been shown that the V4 region is less able to capture the skin microbiota when compared to e.g. the V1–V3 region^26^.

In conclusion, this study is the first to demonstrate that the cutaneous microbiome of the unaffected skin differs in patients with sacral pressure ulcers from those without pressure ulcers. In the future, this might offer a novel method to improve assessment of patients at risk for development of pressure ulcers using the cutaneous microbiome as a biomarker.

## Methods

### Participants

We conducted a single centre, cross-sectional, case-control study at the Maastricht University Medical Centre (MUMC+). The study was approved by the medical ethics committee of the MUMC+ and written informed consent was obtained from all the study participants. The study was conducted in compliance with ethical rules for human experimentation that are stated in the Declaration of Helsinki. A total of thirty patients were able to participate at this study; 15 patients with a hospital acquired category ≥2 sacral pressure ulcer (decubitus group, DC) and 15 patients with no (pre-)existing pressure ulcers (control group, NoDC). All patients had to be bound to their bed or chair admitted to one of the wards of the MUMC+.

Exclusion criteria were: age <18 years, mentally disabled patients/unable to give informed consent, any skin diseases/wounds/signs of infection at the skin of vertebrae level L3.

### Hygiene and treatment of pressure ulcers

In our hospital, immobile patients (all study participants) were washed one time a day in the morning. This was done by bed bath using soap, towels, water, and washcloths. There was no difference in washing procedures in patients with or without pressure ulcers. Standard pressure ulcer treatment consisted of an anti-decubitus mattress and repositioning. The local treatment of pressure ulcers consisted of the application of a foam wound dressing in ten patients. Three patients had anti septic crème applied on the pressure ulcer underneath their wound dressing. Two patients had a vacuum-assisted closure system on their pressure ulcer. One patient received barrier crème between his buttocks. One patient received no extra local treatment.

### Sample collection

To study the cutaneous microbiome of the unaffected skin, skin samples were obtained using Copan FloqSwabs^TM^ (Copan Flock Technologies, Brescia, Italy) under sterile conditions. First, the skin of vertebrae level L3 was inspected for any signs of infection (erythema), skin diseases or wounds. When the skin was unaffected, an area of 4 cm^2^ was marked with a skin marker. The swab was pre-moistened with Reduced Transport Fluid (RTF) buffer^27^. Then, the skin was stretched and swabbed 50 times back and forth applying firm pressure. After swabbing, the tips were placed in a vial containing 50 µl of RTF buffer and immediately placed on dry ice before storage in a −80 °C freezer until further processing. All samples were collected between 01.00–03.00 PM. To prevent bias due to variation in sample collection, all skin samples were obtained by one single researcher.

Subsequently, sex, age, length, weight, medical diagnosis, co- morbidities, antibiotics use, pressure ulcer category, and pressure ulcer treatment were recorded for each patient.

### DNA isolation

After adding 180 µl lysis buffer (20 mM Tris pH8, 2 mM EDTA pH8, 1.2% Triton X-100) with lysozyme (20 mg/ml), swabs were incubated for 30 min at 37 °C and subsequently mixed by vortexing. After adding 200 µl Buffer AL and 25 µl Proteinase K from the QIAamp DNA mini kit (Qiagen, Hilden, Germany) and mixing by vortexing, samples were incubated overnight at 56 °C. The next day, 200 µl of ethanol (96–100%) was added and mixed by vortexing the complete lysate. Subsequent steps were conducted according to the protocol of the QIAamp DNA mini kit as per manufacturer’s instructions, except that the DNA was eluted in a final volume of 100 µl.

### Sequencing

Amplicon libraries and sequencing was performed according to previously published protocols^27^. Briefly, the V4 region of the 16S rRNA gene was PCR amplified from each DNA sample in triplicate using the 515 f/806r primer pair^28^. Pooled amplicons from the triplicate reactions were purified using AMPure XP purification (Agencourt, Massachusetts, USA) according to the manufacturer’s instructions and eluted in 25 µl 1 × low TE (10 mM Tris-HCl, 0.1 mM EDTA, pH 8.0) and subsequently quantified by Quant-iT PicoGreen dsDNA reagent kit (Invitrogen, New York, USA*)* using a Victor3 Multilabel Counter (Perkin Elmer, Waltham, USA). Amplicons were mixed in equimolar concentrations to ensure equal representation of each sample and sequenced on an Illumina MiSeq instrument.

### Data analysis and statistics

The V4 16S rDNA bacterial sequences generated in the present study were deposited in QIITA archive (accession number: 11145). Filtering, denoising, removing of chimeric sequences, and clustering of sequences in Operational Taxonomic Units (OTUs) at 97% similarity was conducted using the LotuS (version 1.39) pipeline^29^. First, sequences with an average quality below 27, read length below 170 bases, one or more ambiguous bases, or containing homopolymer stretches of over 8 bases were discarded for further analysis. Retained sequences were chimera filtered and clustered into OTUs with UPARSE^30^. Taxonomic annotation of OTUs was derived from RDP naïve bayes classifier annotations (minimum acceptance confidence set at 0.8)^31^.

Singletons and OTUs with less than 10 reads were removed. QIIME version 1.8.1^32^ and R version 3.1.3 were used to conduct downstream analyses.

#### Assessing differences on a species level

To examine potential differences in the relative abundance of bacterial species between pressure ulcer patients (DC) and control patients (NoDC), all OTUs that were taxonomically assigned to the same species were combined. We evaluated different methods for normalization and abundance testing by the R tool DAtest^33^ (Supplementary File 1). For all subsequent analyses, we normalized the count-table using variant stabilization by the R-package DESeq. 2^34^. The number of sequence reads ranged from 29,073 to 258,211 species counts in one sample. We used size factor correction to account for these differences in sequencing depth between the samples. DESeq. 2 was also applied to obtain dispersion estimates and to test for differential abundance of each species between pressure ulcer patients (DC) and control patients (NoDC) using default parameters. Results are reported as log2 fold changes and associated adjusted p-values (BH-correction).

Dendrograms were obtained by hierarchical clustering using Ward’s method where Pearson’s correlation was used as the distance measure.

#### Microbial alpha and beta diversity

The following metrics of species richness and diversity within communities (alpha-diversity) were determined: Observed OTUs (observed richness), Chao1 index (estimated richness), Shannon index (estimated diversity) and Good’s coverage. Normality was tested with the D’agostino & Pearson omnibus test. The Mann Whitney U test was used to compare the alpha diversity metrics among the patients in the DC group to the patients in the NoDC group.

The dissimilarity in the microbial community composition (beta-diversity) between each pair of skin samples was assessed using the Bray-Curtis and (un)weighted UniFrac distance^8^ and Bray-Curtis dissimilarity (BC). Clustering of samples was visualized using Principal Coordinate analysis (PCoA). Separation of pressure ulcer patients and control patients was tested by means of permutational multivariate analysis of variance on both the Bray-Curtis and weighted Unifrac distance matrixes using the Adonis function as implemented in the R package vegan using 1000 permutations. Nonparametric two- sample t-tests with Bonferroni correction based upon 1000 Monte Carlo permutations were used to compare the beta-diversity within pressure ulcer patients (average pairwise distance between all samples in the DC group) to the beta diversity within the control patients (NoDC) (average pairwise distance between all samples in the NoDC group) and the between group beta-diversity (average pairwise distance between samples of each patient in the DC group and each NoDC group patient).

#### Machine learning

Random forests are an effective approach for analyzing and interpreting high-dimensional data. The method correlates species abundance or clinical data with pressure ulcer occurrence and introduces a novel bivariate node-splitting. We used the R package randomForestSRC^35^ and the Boruta algorithm for feature selection. The bootstrapped feature selection was repeated 1000 times with differing random seeds. A number of trees of 500 was found to produce a stable error rate.

## Supplementary information

Supplementaryinformation
