# Supplementary material for: The cutaneous microbiome in hospitalized patients with pressure ulcers
Source: Sci Rep. 2020 Apr 6;10:5963. doi: 10.1038/s41598-020-62918-8 (PMC7136253; doi:10.1038/s41598-020-62918-8)
Supplement: Supplementary file 1 — Supplementaryinformation [file 41598_2020_62918_MOESM1_ESM.pdf]

Supplementary File 1
The cutaneous microbiome in hospitalized patients with pressure ulcers

Luuk A. de Wert[1,2], Sander S. Rensen[1,2], Zita Soons[1,2,*], Martijn Poeze[1,2], Nicole.D. Bouvy[1,2], John Penders[2,3,4]

1. Department of Surgery, Maastricht University Medical Centre, Maastricht, The Netherlands
2. NUTRIM School for Nutrition and Translational Metabolism, Maastricht University, Maastricht, The Netherlands.
3. Department of Medical Microbiology, Maastricht University Medical Centre, Maastricht, The Netherlands
4. CAPHRI School for Public Health and Primary care, Maastricht University Medical Centre, Maastricht, The Netherlands

*Corresponding author, Zita Soons; zita.soons@maastrichtuniversity.nl, Maastricht, The Netherlands, Universiteitssingel 50, P.O. Box 616, 6200 MD Maastricht, Phone +31 433881992, Fax +31 433884154.

Normalization and differential abundance testing

We aimed to use the package most suitable to our data. This choice was not straightforward, since the performance of the packages, in terms of for instance AUC, is not known on real-life data. To gain insight in performance of the packages we have evaluated 25 methods for normalization and abundance testing by the R tool DAtest (https://github.com/Russel88/DAtest)[1]. We tested all packages that were available in R version 3.3.0 and present our findings and motivation for the package of choice below.

The Venn Diagram in Supplementary Figure 1 shows that the results (species with differential abundance) are dependent on the chosen method.

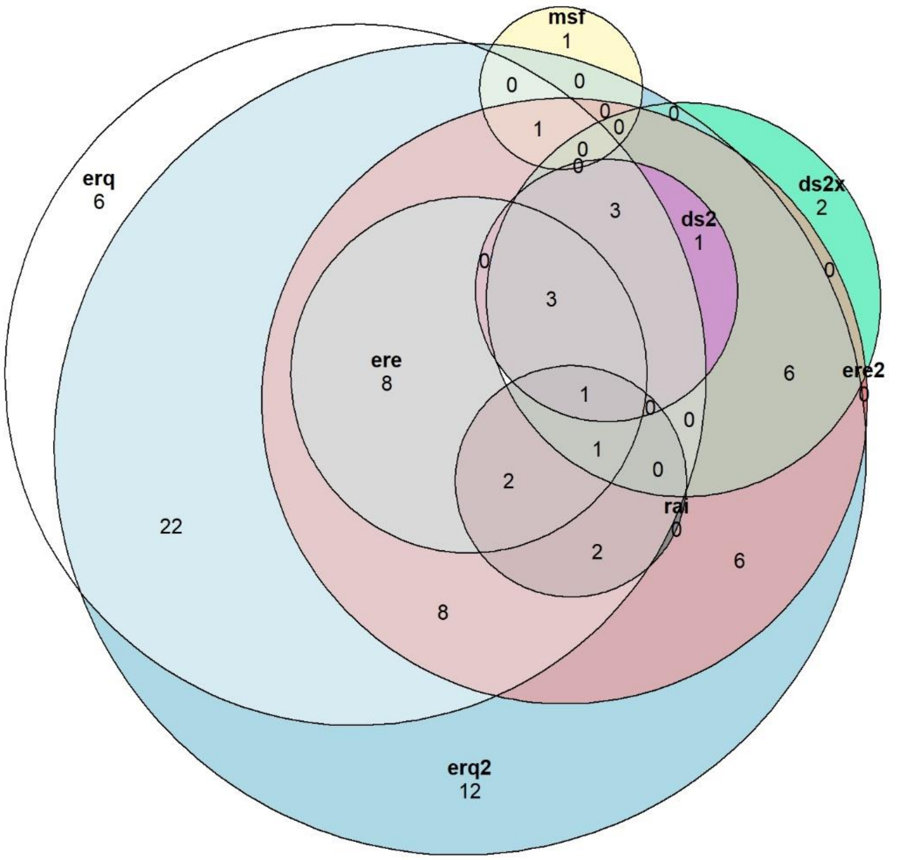

Supplementary Figure 1. Venn diagram for significant differential abundances for the tested methods (adjusted p-value<0.05) using the pressure ulcer dataset.

Considering the subsequent choice of the method, we avoided packages using rarefying (commonly done on microbiome data) because of a loss of sensitivity due to elimination of part of the data. RAIDA and MetagenomeSeq assume that the log-transformed data follow a normal distribution and ALDEx2 a Dirichlet distribution, which are continuous distributions. Since RNAseq counts of species represent discrete data and contain many zeros (shown in Supplementary Figure 2 using the

pressure ulcer dataset), their analysis requires the use of a proper mathematical theory. We hence believe a Poisson or negative binomial distribution is more appropriate to model the data.
BaySeq assumes a negative binomial distribution, but performance was found highly variable in Seyednasrollah *et al.*[2].
DESeq2 and EdgeR model the discrete nature of count data. EgdeR identified a considerable amount of species with differential abundance (ere, ere2, erq, and erq2 in Supplementary Figure 1), but is known to suffer from a high false discovery rate. DESeq(2) uses the same normalisation method as edgeR, but is more conservative leading to a lower false discovery rate. This can also be seen from the lower number of species with differential abundance in the Venn diagram in Supplementary Figure 1. In addition, Weiss et al.[3] showed increased sensitivity of DESeq2 using simulated datasets with fewer than 20 samples per group (15 per group in the pressure ulcer dataset).
Concluding, DESeq2 is the preferred choice to test differential abundance.

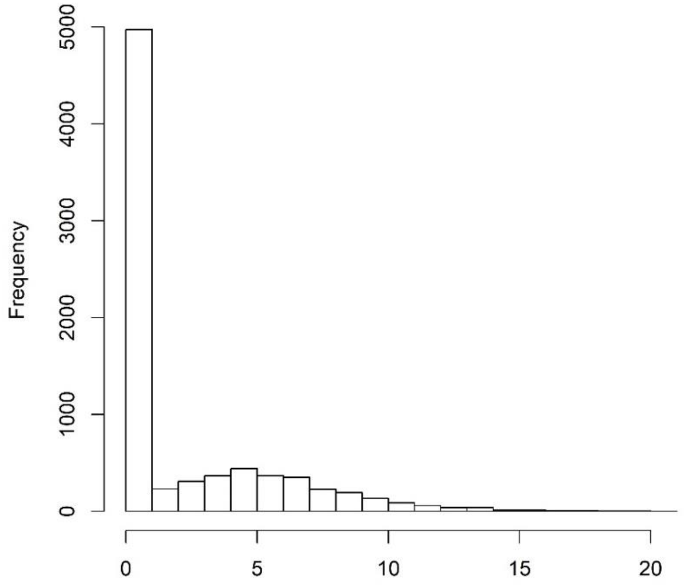

Supplementary Figure 2. Histogram of log2 counts.

References

1 Russel J, *et al*. DAtest: a framework for choosing differential abundance or expression method. *bioRXiv*, doi: https://doi.org/10.1101/241802 (2018).
2 Seyednasrollah, F., Laiho, A. & Elo LL. Comparison of software packages for detecting differential expression in RNA-seq studies. *Brief Bioinform* **16**, 59-70 (2015).
3 Weiss S *et al*. Normalization and microbial differential abundance strategies depend upon data characteristics. *Microbiome* **5**, 27 (2017).

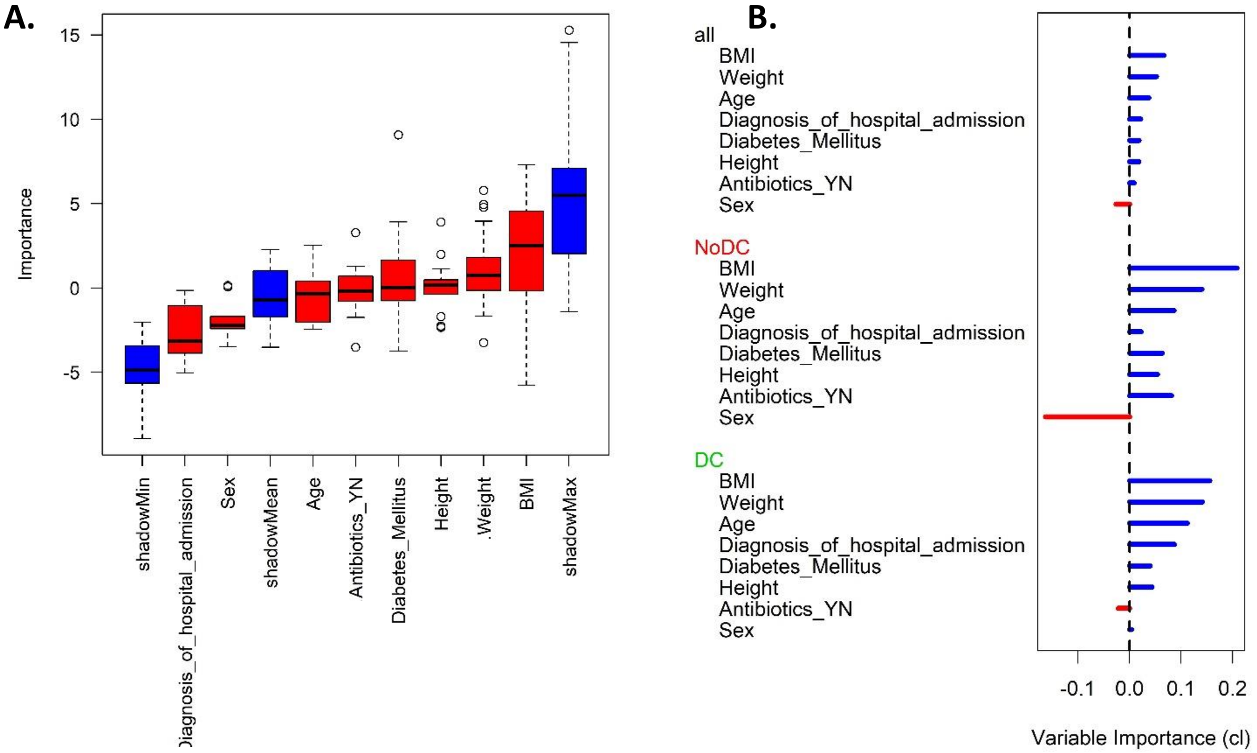

C.

```
         predicted
observed NoDC DC class.error
    NoDC     6  6      0.5000
    DC       5 10      0.3333

      Overall error rate: 40.74%
```

Supplementary figure 3. Random forests with Boruta feature selection using clinical data. **A**. The importance of each feature across 1000 repeats is shown compared with the worst, best and mean randomly generated features. **B**. Variable importance in the random forest. **C**. Confusion matrix.
